# Design of a novel filter paper based construct for rapid analysis of acetone

**DOI:** 10.1371/journal.pone.0199978

**Published:** 2018-07-06

**Authors:** Sajid Rauf, Yaqeen Ali, Sabir Hussain, Fakhar Ullah, Akhtar Hayat

**Affiliations:** 1 Interdisciplinary Research centre in Biomedical Materials (IRCBM), COMSATS Institute of Information technology, Lahore, Pakistan; 2 Department of Computer Science, COMSATS Institute of Information technology, Lahore, Pakistan; 3 Department of Computer Science and Information Technology, University of Sargodha, Sargodha, Pakistan; Institute of Materials Science, GERMANY

## Abstract

The present work was focused to design a cheap, rapid, portable and easy to use filter paper based assay for the qualitative and quantitate analysis of acetone. Sodium alginate gel was loaded with the acetone specific optical signal probe, and subsequently coated onto filter paper surface to design portable colorimetric assays for acetone monitoring. The color of the paper sensor strip was observed to change from dark yellow to light yellowish in the presence of varying concentrations of acetone. Three different color analyzing models including RGB, HSV, and LAB were employed to probe the output optical signal, and their performance was compared in terms of better interpretation of the generated signal. The LAB model was found to provide better analytical figures of merit with a linear response for the acetone concentration ranging from 2.5 to 1500 ppm, and a limit of detection of 0.5 ppm. Furthermore, the specificity of the designed filter paper based sensor was demonstrated against different common interfering compounds. The results demonstrated the potential of our proposed filter paper based sensor as a novel tool for the analysis of acetone.

## 1. Introduction

Human breath contain hundreds of different types of volatile organic compounds (VOCs) which are exhaled from blood into lungs [1, 2]. The precise assessment of concentrations of VOCs offers valuable information that can be further used as markers to analyze the human body health condition. The use of exhaled breath to diagnose different diseases has attracted the attention of researchers due to advantages like, easy and non-invasive testing. Among all these VOCs, acetone is one of the compound that can serve as a marker to provide information on the status of patients suffering from diabetes [3]. It has been established that acetone concentration in healthy human is 0.35–0.85 ppm, while in case of diabetic patient increased to 2–2.25 ppm [4, 5]. Acetone has been emerged as a more sensitive breath marker towards control of diabetes in comparison to assays based on the blood glucose monitoring [6]. Therefore, breath acetone testing plays an important role in monitoring the diabetes as well to control the intake of insulin for diabetics patients [7]. The main source of acetone exhaled by human is from decarboxylation of excess acetyl-coenzyme. Breathe acetone analysis can play a vital role in diagnosing diabetes type-I and type-II [8, 9].

Generally, the analysis of breath is facing many challenges in terms of designing a non-invasive and portable device for rapid and cost effective measurement of different breath markers especially, acetone marker. Analytical methodologies such as optical spectroscopy [10], gas chromatography/mass spectroscopy (GC/MS) [1, 2, 11], and selected ion-flow tube–mass spectrometry (SIFT–MS) [12] have been widely employed for breath analysis to diagnose asthma [13], heart disease [14], lung cancer [1], kidney disorders [15] and diabetes [16]. These analytical techniques have shown high selectivity, sufficient sensitivity and low limit of detection for several VOCs. However, there are many limitations in the above methods including complicated measurement methodologies, need of sophisticated equipment and highly trained persons to operate, elevated cost per assay [17]. Many authors have reported different gas sensors exploring composite oxide materials for detection of various analytes. Some of the examples include detection of trimethylamine based on ZnO:Cr_2_O_3_ [18], detection of ammonia (NH_3_) based on Fe_2_O_3_:ZnO nanocomposite [19], detection of NO_2_ based on nanocomposite WO_3_:SnO_2_ [20], H_2_S detection based on CuO:SnO_2_ [21], detection of formaldehyde based on In_2_O_3_:ZnO nanocomposite [22], and detection of NO_2_ based on ITO:ZnO nanocomposite [23]. The selectivity of a sensor is of vital importance to provide more accurate and precise information of a given analyte in a particular matrix. Various metal oxides based sensing platforms have been reported to enhance the selectivity of gas sensors [24–26]. Similarly, the development of gas sensors based on hierarchical metal oxides, binary metal oxides can pave a novel way for highly sensitive and selective analysis of gases[27–30]. In this direction, metal oxides such as ZnO [31, 32], copper oxide [33], indium oxide [34], titanium dioxide [35], WO_3_ [36], MoO_3_ [37] and α-Fe_2_O_3_@SnO_2_ core–shell heterostructure nanotubes [38] have been investigated for the detection of acetone. Despite of all this progress, it is highly desirable to design portable, economical and robust assays.

Alternatively, this work is focused to develop a filter paper based acetone sensor. The filter paper sensors are portable, easy to handle, economical, do not require special temperature conditions, do not need complex instrumentation and can be used in the remote areas. Whatman® filter paper No. 1 was used in the present work which offers medium retention and flow rate. The main constituent of filter paper is cellulose fiber and which allows liquid to penetrate within its hydrophilic fiber matrix without need of an active pump or external source. Sodium alginate gel is another constitute of our sensing system. Sodium alginate is well known for its adsorption properties and high porosity [39]. Sodium alginate gel was loaded with the acetone specific optical signal generating reagents, and was further immobilized onto filter paper based transducer surface to design portable colorimetric assays for acetone monitoring. Three different color analyzing models including RGB, HSV, and LAB were employed to probe the output optical signal and their performance was compared in terms of better interpretation of the generated signal.

## 2. Materials and methods

### 2.1 Reagents

Sodium hydroxide NaOH, 4-aminobenzoic acid ( ≥99%), hydrochloric acid HCL (35–37%), acetic acid CH_3_COOH (99.9–100.5%), acetone, sodium nitrite ( ≥97.0%), sodium alginate C_6_H_9_NaO_7_ and filter paper (Whatman grade 1) were purchased from Sigma-Aldrich. Stock solutions of analyte were prepared in acetic acid. All of the above reagents were of analytical grade and used without purification. All of the solutions were prepared in deionized water from ELGA PURELAB® Ultra water deionizer.

### 2.2. Apparatus and characterization

UV−Vis absorption spectra were recorded using a double beam Perkin Elmer UV-Vis spectrophotometer Lambda-25 (UV-25, Perkin Singapore) with 10 mm disposable cuvettes having 2 ml capacity and a bandwidth setting of 1 nm at a scan speed of 960 nm/min in the range of 370 to 700 nm.

### 2.3. Preparation of diazotized 4-aminobenzoic acid

The preparation of diazotized 4-aminobenzoic acid was carried out according to a procedure described by Bashir and his co-workers [40]. Briefly, 0.3 gm of 4-aminobenzoic acid was added into the 20 mL of deionized water containing 0.75 mL hydrochloric acid (HCL) and shacked for 1 min to get homogenous solution. In the same manner, 0.45gm of sodium nitrite (NaNO_2_) was added into 5 mL of deionized water. Both solutions were placed at a low temperature of -10 ^0^C for 5 min. Subsequently, both the solutions were mixed to obtain the diazotized 4-aminobenzoic acid. In the same manner, NaOH solution was prepared by adding 8 gm of NaOH into 12 ml of deionized water.

### 2.4. Spectrophotometric analysis of acetone

Spectrophotometric determination of acetone with the prepared diazotized 4-Aminobenzoic acid was performed by mixing NaOH (15.5 mM), 300 μL of diazotized solution and adding acetone (0.25 M in serial dilution) into de-ionized water to obtain the final acetone concentrations ranging from 0.25 to 1.5 mM. The absorbance was measured from 370 to 700 nm with 10 mm disposable cuvettes having 2 mL capacity and a bandwidth setting of 1 nm at a scan speed of 960 nm/min through Perkin Elmer UV-Vis spectrophotometer Lambda-25.

### 2.5. Preparation and loading of sodium alginate gel

In order to prepare and load the sodium alginate gel with acetone specific reagents, 300 μL of the prepared diazotized 4-aminobenzoic acid and 200 μL of NaOH solution was added into 9.5 mL of deionized water. Then, 1 gm of sodium alginate was added into the above solution gradually and stirred the mixture at 350 rpm at 80 ^o^C in order to avoid precipitation and to get the homogenous gel.

### 2.6. Preparation of sodium Alginate-Based filter paper

Round-shaped Whatman no. 1 filter paper were cut with a hole-puncher and soaked in sodium alginate gel loaded with acetone specific reagents and used to achieve the sodium alginate gel-based filter paper surface. Subsequently, various concentrations of acetone were incubated for optimized time period of 2 min, and dried at 45 ^**o**^C for 30 min. The diameter of the strip was 0.6 cm.

### 2.7. Filter paper based analysis of acetone

In order to investigate the filter paper based analysis of acetone, the sodium alginate gel was loaded with acetone specific reagents, and subsequently coated onto Whatman no1 filter paper surface to design portable colorimetric assays for acetone monitoring. The color of the paper sensor strip was observed to change from dark yellow to light yellowish upon incubation of different concentrations of acetone. To analyze the output optical signal and their performance, three different color analyzing models including RGB, HSV, and LAB were employed. Calibration curve was constructed to determine the analytical performance of the colorimetric assay based on the interpretation of the color analyzing model.

### 2.8. Color intensity measurement

The color of loaded sodium alginate based modified paper strip was altered by the addition of acetone. In this manner, for quantification of the color, the acetone-based sodium alginate paper strips were scanned with office used scanner. The images obtained with scanner were analyzed using the Adobe Photoshop. Three different color analyzing models, RGB, HSV, and LAB were employed and compared to analyze different concentrations of acetone on filter paper. All these three models are based on different analyzing approach with different functionalities.

#### 2.8.1 RGB (red, green and blue) color model

The RGB color model consists of three additive primary colors including red, green and blue. The verity of colors can be produced by mixing these colors in different ways, depending on how much is taken from each base color. The theory behind RGB model is based on human perception of colors. RGB color model is used in televisions, computers, graphics cards and monitors or LCDs to represent and display images. A color image can be formed by making three measurements of scene brightness at each pixel, using the red, green and blue components of the detected light.

#### 2.8.2 HSV color space

HSV is named based on three values—Hue, Saturation and Value. The HSV is a perceptual color space mostly expressed as a non-linear combination of the RGB values. HSV is often called “hexcone model". Saturation refers to the intensity of color in an image. In technical terms, it is the expression of the bandwidth of light from a source. The term hue refers to the color of the image itself, while saturation describes the intensity (purity) of that hue and value referred to "brightness". The transformation from RGB to HSV is given by equations (1)(2)(3):

$$H=accros\frac{\frac{1}{2}(2R-G-B)}{\sqrt{{\left(R-G\right)}^{2}-(R-B)-(G-B)}}$$ (1)

$$S=\frac{max\;(R,G,B)-min\;(R,G,B)}{max\;(R,G,B)}$$ (2)

V=max(R,G,B) (3)

#### 2.8.3. Lab color space

LAB is a three-dimensional one channel color space for Luminance (lightness) L, along with two color channels a and b. The most important feature of Lab is device independence, and the colors are defined independent of their nature of creation or the device they are displayed on. The L channel has values ranging from 0 up to 100, which corresponds to different shades from black to white. The a channel has values ranging from −128 up to +127 and gives red to green ratio. The b channel also has values ranging from −128 up to +127 and gives yellow to blue ratio. Thus, a high value in a or b channel represents a color having more red or yellow, while a low value represents a color having more green or blue. Delta E is the distance between two colors in LAB color space. Delta E2000 is an advanced algorithm version of this concept, and therefore is employed in the given study to provide improved accuracy to differentiate the colors.

## 3. Results and discussions

### 3.1. Principle of the assay

**Fig 1:**
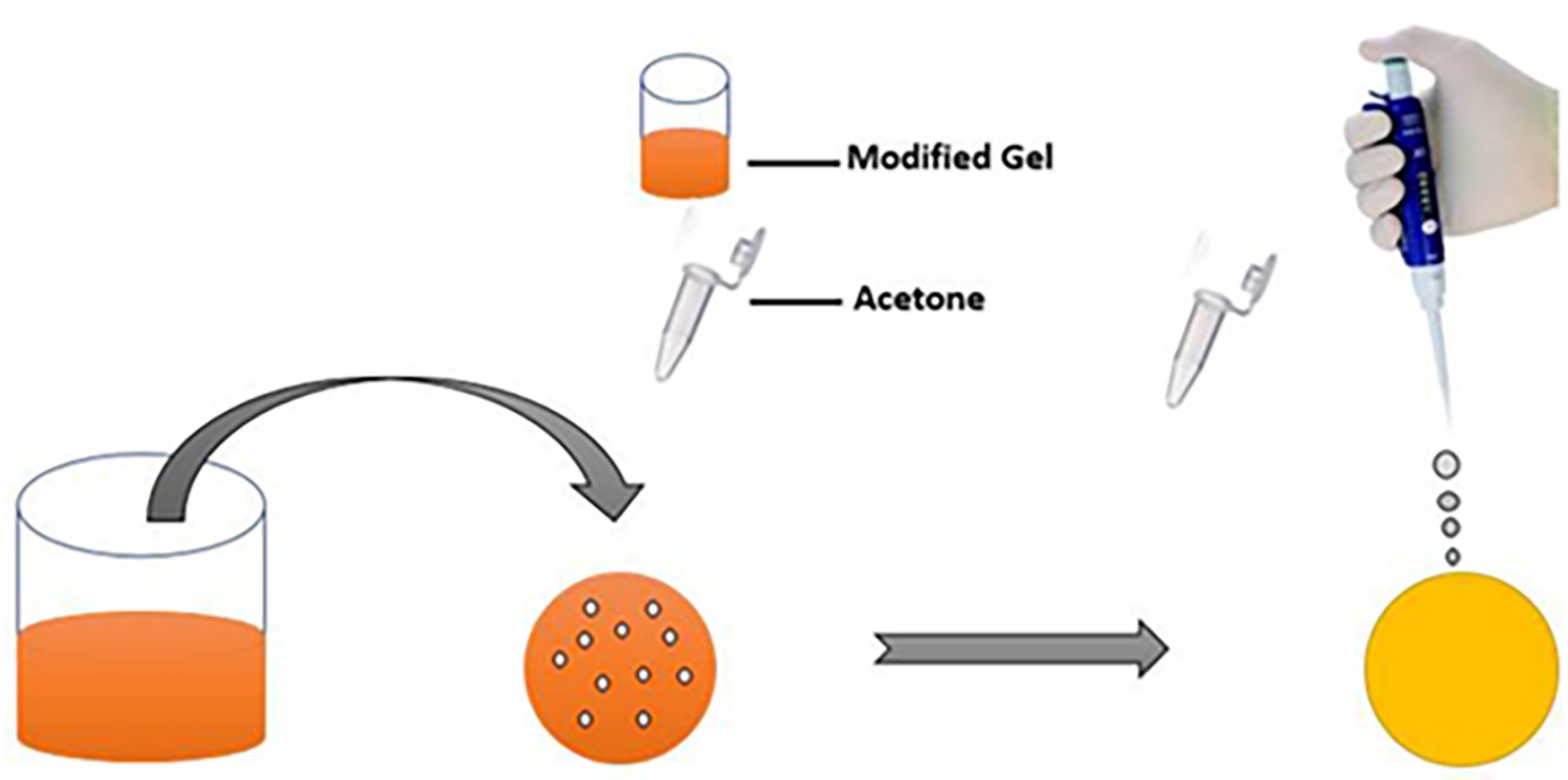
Schematic depiction of the assay principle; coating of sodium alginate gel loaded with the acetone specific optical signal probe on filter paper/change in the optical properties of modified filter paper upon incubation with acetone.

The working principle of the modified sodium alginate gel based filter paper for the detection of acetone is presented in Fig 1. Acetone specific optical probe loaded sodium alginate gel was immobilized onto a filter paper platform, and the single step was performed to analysis the addition of the analyte. Subsequently, modified filter paper interacted with acetone on exposure and alteration in the color was observed. The assay principle was based on the reaction of acetone with diazotized *p*-aminobenzoic acid in a strongly alkaline medium. The diazotized *p*-aminobenzoic acid loaded alginate gel/solution in the presence of alkaline medium was characterized with a dark purple color upon incubation with acetone. The intensity of the generated color was dependent on the centration of acetone. This correlation between color intensity and acetone concentration was employed for the quantitative analysis of the acetone. Spectrophotometer analysis were performed for assay in solution, while filter paper based assays were based on the analyzing models.

### 3.2. Spectrophotometric determination of acetone

Initially, the interaction of prepared acetone specific optical probe organic compound diazotized 4-amino benzoic acid with acetone was investigated. The color reaction was very fast and the procedure was simple. The diazotized solution generated intense dark purple color in aqueous solution in the presence of acetone as an analyte using NaOH with strongly distance-dependent optical properties. Control experiments in the absence of acetone specific reagents, alkaline medium NaOH and acetone were performed in order to ensure the feasibility of the method for acetone analysis. The control experiments illustrated that absorbance was very negligible for individual component as compared to the absorbance of all components of the sensing system (S1 Fig).

#### 3.2.1 Optimization of experimental conditions

In order to achieve the optimal performance for the spectrophotometric determination of acetone, the effects of alkaline medium and volume of diazotized 4-amino benzoic acid were optimized. The color change and absorbance of this sensing system were dependent on the mentioned variable (S2 Fig). The highly sensitive response was obtained under following optimal conditions: pH 7.4, 300 μL of diazotized 4-amino benzoic acid and 15.5 mM NaOH based alkaline medium at room temperature and the color reaction was spontaneous. The optimum amount of NaOH solution was also used to stabilize and retain the color for a maximum time period (S2 Fig).

#### 3.2.2 Detection of acetone

**Fig 2:**
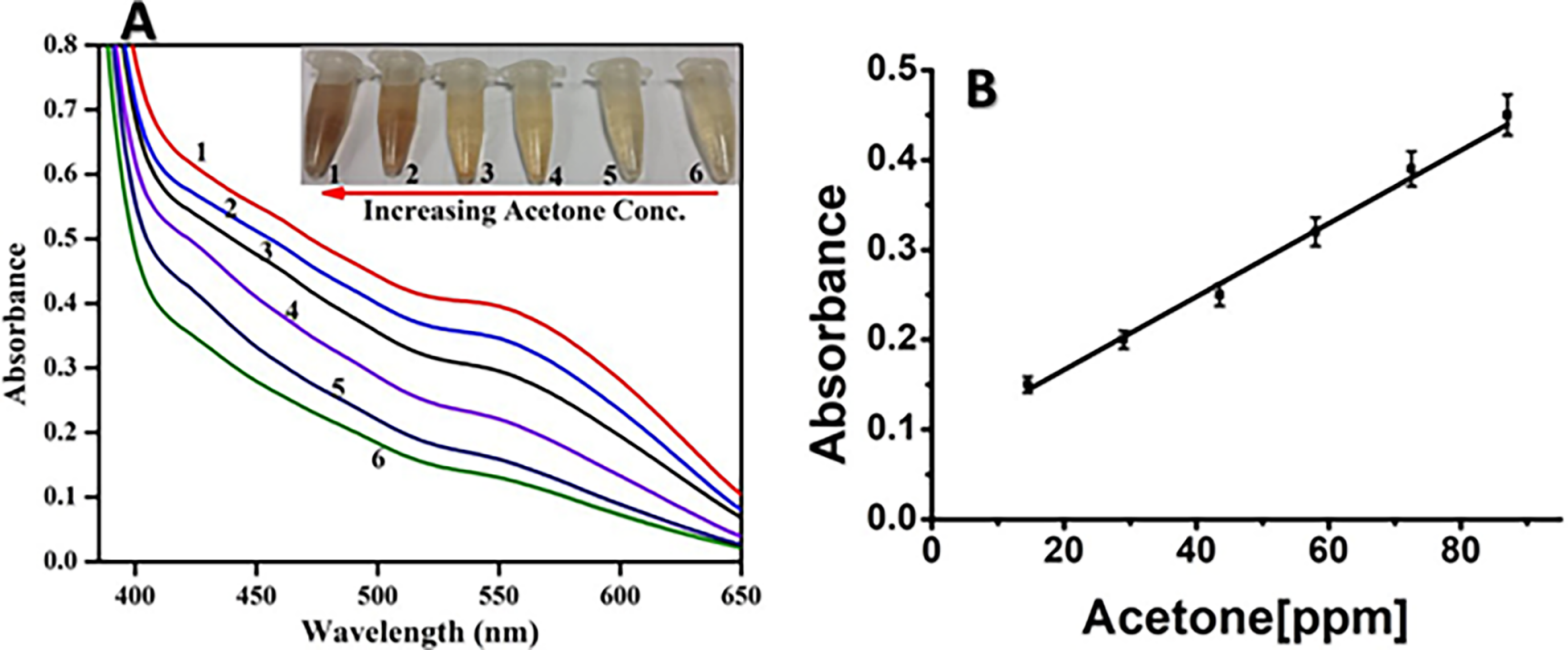
UV/Vis response of the varying concentrations of Acetone (mM), (1) 1.5, (2) 1.25, (3) 1, (4) 0.75, (5) 0.5, (6) 0.25, inset shows the corresponding visual changes in color (A); Corresponding calibration curve in terms of (ppm) 14.5–87 (B).

Under the optimum experimental condition, the spectrophotometric determination of acetone was performed based on the color change, and the absorbance of diazotized solution upon incubation with acetone. This sensing system was linearly dependent on acetone concentration. Keeping in view the color change, a quantitative calorimetric method was developed based on the relationship between acetone concentration and absorbance intensity as showed in Fig 2A. The generated output optical signal was linearly dependent on the concentration of acetone, and the method enabled the detection of acetone with a linear range of 0.25–1.5 mM. The standard curve is plotted based on the absorbance as a function of acetone concentration to detect the strong optical changes in Fig 2B. It is noteworthy that the proposed method offers a strategy to monitor acetone by recording the clear visual change in the dark purple color which is completely distinguishable by the naked eye.

### 3.3. Filter paper based assay

**Fig 3:**
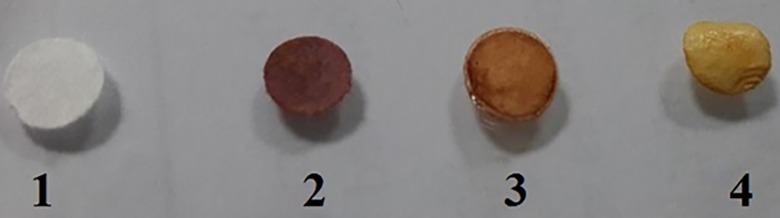
Optical inset for filter paper based assay in the presence of control in the presence of acetone (0.12 mM) (1); control in the presence of 5 μL of diazotized 4-aminobenzoic acid solution (2); control in the presence of acetone specific reagents loaded sodium alginate gel (0.2 g/mL) and acetone (0.12 mM) (3); in the presence acetone specific reagents loaded sodium alginate gel (0.2 g/mL), NaOH (15.5 mM) and acetone (0.12 mM) (4).

To adapt this assay to paper format, we immobilized sodium alginate gel loaded with acetone specific reagents onto Whatman no. 1 filter paper. The method provided an easy and efficient fabrication of filter paper surface with modified sodium alginate gel with good uniformity and surface coverage. The immobilized sodium alginate gel loaded with acetone specific reagents altered their color changing properties in response to acetone. The color of the paper strip was changed from dark yellow to light yellowish after incubation with acetone. The produced color was evenly distributed on the surface of the sensing strip, indicating uniform distribution and surface coverage of the loaded sodium alginate gel onto the paper strip and also confirming the effectiveness of the immobilization process. To prove this concept, different control experiments were performed. Similarly, various control experiments were demonstrated in the absence of acetone specific reagents, alkaline medium NaOH and acetone. Fig 3 shows the distinguishable response of the paper assay upon incubation of acetone as compared to the control experiments.

#### 3.3.1 Filter paper based assay optimization

Prior to acetone detection, the filter paper based assay was optimized for experimental conditions such as concentration of sodium alginate in gel preparation, incubation time of soaking filter paper strip in sodium alginate gel loaded with specific reagents, and incubation time of acetone on modified sensing strip. The optimum concentration of sodium alginate to prepare gel was 0.2 g/mL and incubation time of soaking filter paper strip in modified sodium alginate gel was 1.5 min (S3 Fig). The higher concentrations of sodium alginate in gel was found to rapture the paper surface. Optical signals generated were evaluated for varying incubation times (1, 2, 4, 6 and 10 min) of acetone. Optimal incubation time of acetone was 2 min reaching equilibrium (S3 Fig).

#### 3.3.2 Detection of acetone

**Fig 4:**
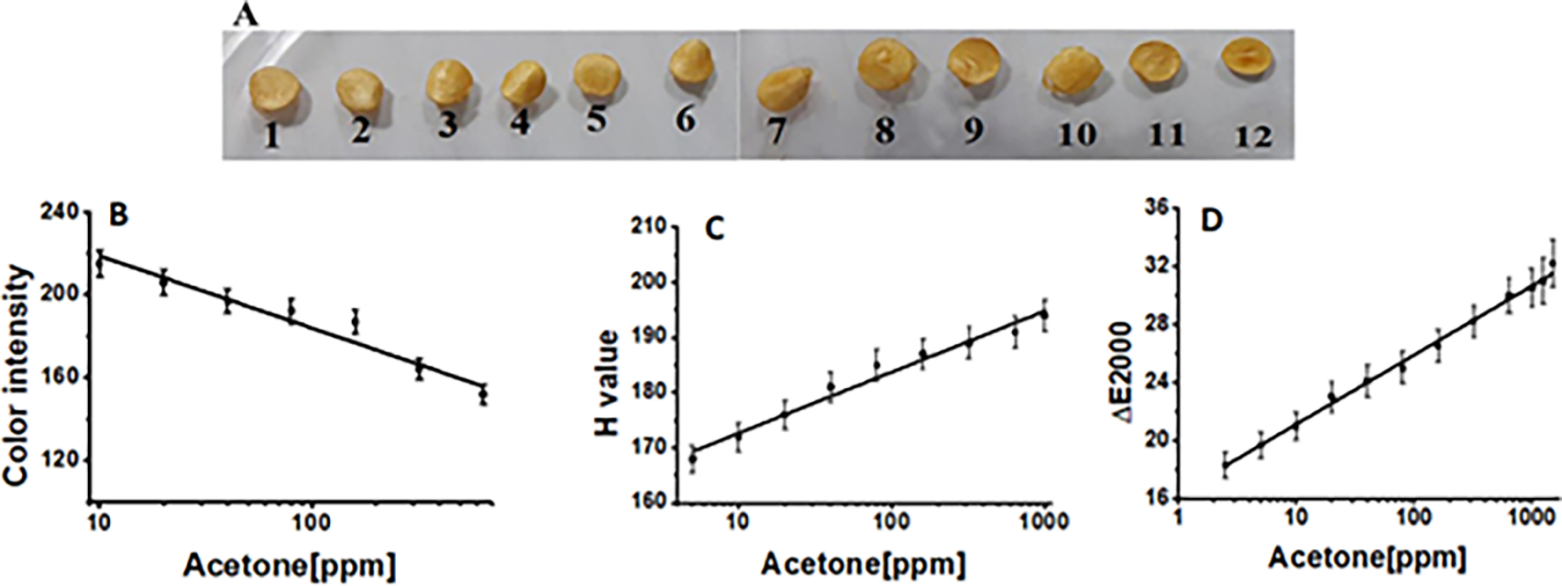
Modified sodium alginate gel-based paper assay for the detection of acetone. Colorimetric responses of Modified sodium alginate gel-based paper strips to concentrations of acetone ranging from 0.12 mM to 0.25 M, i.e. (1) 0.25 M, (2) 0.125M, (3) 0.0625 M (4) 31.25 mM, (5) 15.625 mM, (6) 7.812 mM, (7) 3.91 mM, (8) 1.95 mM, (9) 0.97 mM, (10) 0.49 mM, (11) 0.24 mM, (12) 0.12 mM (A); Linear calibration curve for acetone based on color analyzing models RGB (B); HSV (C); LAB (D).

Acetone is one of the important VOC inside body and can serve as a marker to provide information on the status of patients suffering from diabetes. The colorimetric filter paper based sensor for the detection of acetone with a simple procedure, short analysis time, small volume and low cost, was conveniently realized by coating the sodium alginate gel loaded with acetone specific optical signal probe on filter paper strip. Subsequently, modified paper sensor strip in the presence of different concentration of acetone visually changed the color from dark yellow to light yellowish in a concentration-dependent manner, and was dried at temperature of 45 ^o^C. The calibration curves were obtained by the better interpretation of the generated color with the color analyzing models. Table 1 summarizes the analytical performance in terms of linear range and limit of detection for three color analyzing models. As can be seen from the Table 1 and Fig 4, better analytical characteristics were achieved with LAB analyzing model. Furthermore, S1, S2 and S3 Tables present the sensing data showing relation between the output color intensity and the input acetone concentration. This method permitted to detect acetone in the linear range from 2.5–1500 ppm with a limit of detection of 0.5 ppm. A wide linear range and a low limit of detection indicates better analytical performance of a given method. Although, presence of acetone at 1500 ppm level is not common especially in human breath, however, the objective was to demonstrate the wide linear range response of the sensor. Similarly, our method allowed to detect acetone at very low limit of detection.

**Table 1:** Summary of regression plots obtained for three color analyzing models.

*Color analyzing Model*	*Linear Range (ppm)*	*LOD (ppm)*	% RSD^*^	*Correlation Coefficient (R)*
***RGB***	10–640	7.5	5.92	0.99
***HSV***	5–1000	3	4.3	0.97
***LAB***	2.5–1500	0.5	3.5	0.985

*Correspond to replicated values (n = 3).

#### 3.3.3 Interference study

**Fig 5:**
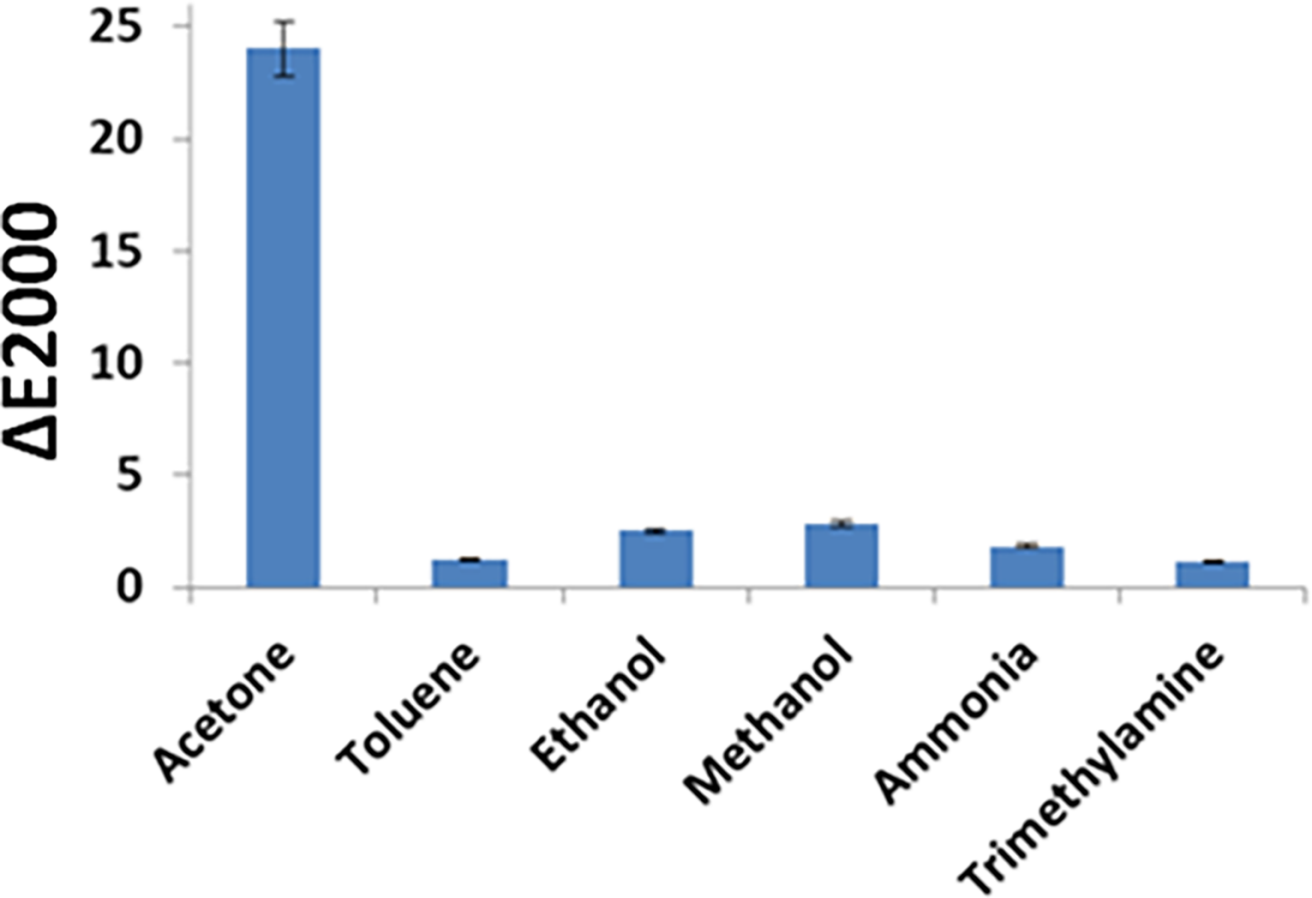
Selectivity analysis of modified sodium alginate gel-based filter paper strip assays for the acetone detection with following analyte concentration; 0.12 mM acetone and 0.24 mM for the rest of interfering compounds.

To continue with the practical applications, the specificity of the proposed method for target analyte is highly desirable. The sensors will be considered reliable if it doesn’t give response to nonspecific compounds. Consequently, the specificity of the designed paper based sensor was evaluated by execution of different control experiments using the nonspecific compounds, like ethanol, methanol, ammonia, trimethylamine and toluene. The modified gel coated onto paper strip was incubated in the presence of nonspecific compounds. The modified paper sensor strip after incubation with interfering molecules didn’t show any color change. From the Fig 5, it can be seen that the obtained values with the color analyzing model for nonspecific compounds are much smaller than that for acetone. The LAB analyzing model based on its better analytical characteristic was employed to investigate the behavior of nonspecific analyte. From these results, it can be concluded that the effect of nonspecific compounds is negligible on acetone detection. Hence, the proposed paper based assay has sufficient specificity to acetone. Furthermore, method reproducibility was examined by evaluating the performance of three independently prepared paper sensors under similar experimental condition upon incubation with same acetone concentration. A standard deviation of 5% was observed for the given experiment, indicating reliable reproducibility of the method. Similarly, when images were scanned with different scanner, a possible human/instrumental error of 5% was observed which is in the range of method reported standard deviation. Furthermore, sensors response was fast requiring an acetone incubation time of 2 min. Our objective was to fabricate cost effective, simple, easy to use, portable and single use assays to give a precise account of the acetone concentration. Keeping in view the cost per assay, it would be economical to employ assay for single to have better and precise results.

Additionally, humidity can play a vital role in human breath analysis. This is an important factor in assay fabrication for the analysis of exhaled breath, which may contain a large amount of water vapor producing a very high humidity level. Such phenomena can causes oxide-based various sensors to not working properly. However, the response of our purposed filter at high humidity (85%) based strip was almost similar to that of observed at room temperature. This response made our sensor strip a very promising tool for use in breath analyzing to detect exhaled acetone.

Compared with other literature reported sensors for acetone analysis, the present filter paper based sensor didn’t require high operating temperature, and involved very easy fabrication steps. The experimental conditions of present designed acetone sensor and other existing acetone gas sensors reported in literature are provided in the Table 2.

**Table 2:** Acetone sensors under ambient environment and temperature.

Materials Used	Environment (Gas)	Optimized operational temperature	Ref
Indium oxide nano wires	Nitrogen	400 ^0^C	[41]
Tungsten trioxide nanoparticles	-	400 ^0^C	[42]
Zinc oxide	-	200 ^0^C	[43]
LaFeO_3_	Air	275 ^0^C	[44]
TiO_2_	Air	500 ^0^C	[45]
InN	Air	200 ^0^C	[46]
Modified Sodium alginate gel fabricated filter paper	Air	45 ^0^C	Present work

Moreover, sensor strip offers advantages of fast response with reduced analysis time, cost effectiveness, portability and does not require special equipment and skilled personal. The sensor strip is highly suitable to perform the on-site analysis without requirement of any specific laboratory. Moreover, the purposed method resulted in very promising analytical figures of merits such as limit of detection, linear range and working temperature. A detailed analytical performance comparison of the purposed method with the recent literature reported detection methodologies is provided in the Table 3.

**Table 3:** Analytical performance comparison of the purposed method with the recent literature reported detection methodologies.

Detection methodology	Limit of detection	Linear range	Ref
Mesoporous In_2_O_3_nanospheres based electrochemical sensor	10	-	[47]
QCM based sensor	3.49	7.05–750	[48]
Cataluminescence sensor	-	5–2500	[49]
Zirconia and CdMoO_4_ based sensing electrode	0.5	5–300	[50]
TiO_2_ nanoparticles functionalized In_2_O_3_ nanowires based sensor	0.8	1.8–10	[51]
PANI/Cellulose/WO_3_ Electrochemical Sensor	10	0–100	[52]
Mixed potential type acetone sensor	0.5	5–500	[53]
Filter paper based sensor	0.5	2.5–1500	Present work

## 4. Conclusion

In conclusion, we have successfully developed of a filter paper assay for the detection of acetone. The proposed chemistry was firstly established through spectrophotometric analysis of acetone and various experimental conditions were optimized. Afterwards, this work has translated the solution chemistry onto a filter paper surface while integrating sodium alginate gel as acetone specific reagent loading and signal generating probe. Our proposed filter paper assay offer advantages in term of portability, low cost per assay, fast analysis time, suitability for on-site analysis and it does not require special equipment or skilled person while comparing to the literature reported methods for acetone monitoring. The high stability, ease in fabrication and reproducibility of the assay combined with the low cost of filter paper transducer surface provide a unique design for the development of highly stable and economical assay for on-site analysis of acetone. The proposed sensor can be an ideal platform for breath analysis in biomedical field.

## Supporting information

S1 Fig Absorption spectra and optical inset for spectrophotometric analysis in the presence of acetone (0.25 mM), NaOH (15.5 mM) and 300 μL of diazotized 4-aminobenzoic acid in 2 mL of de-ionized water reaction medium (T); control in the presence of NaOH (15.5 mM) and 300 μL of diazotized 4-aminobenzoic acid in 2 mL of de-ionized water reaction medium (C1); control in the presence of acetone (0.25 mM) and 300 μL of diazotized 4-aminobenzoic acid in 2 mL of de-ionized water reaction medium (C2); control in the presence of acetone (0.25 mM) and NaOH (15.5 mM) in 2 mL of de-ionized water reaction medium (C3). (TIF)

S2 Fig Optimization of volume of diazotized 4-aminobenzoic acid (μL), (1) 500, (2) 450, (3) 400, (4) 350, (5) 300 in the presence of NaOH (15.5 mM) and Acetone (0.25 mM) in 2 mL of de-ionized water reaction medium **(A)**; Concentration of NaOH solution in the presence of 300 μL of diazotized 4-aminobenzoic acid and acetone (0.25 mM) in 2 mL of de-ionized water reaction medium **(B).**
(TIF)

S3 Fig Optimization of concentration of amount of sodium alginate in gel (g/mL); (1) 0.1, (2) 0.15, (3) 0.2, (4) 0.25, (5) 0.3 **(A)**; time of incubation of dipping filter paper strip to immobilize with modified sodium alginate gel (min); (1) 0.5, (2) 1, (3) 1.5, (4). 2 **(B)**; time of incubation of acetone after addition on modified filter paper strip (min); (1) 1, (2) 2, (3) 4, (4) 6, (5) 10 **(C)**.
(TIF)

S1 Table Sensing data of the output color intensity and input concentration of acetone based on RGB color analyzing model. (DOCX)

S2 Table Sensing data of the output color intensity and input concentration of acetone based on HSV color analyzing model. (DOCX)

S3 Table Actual values of the input concentration and the output color intensity based on LAB color analyzing model. (DOCX)
