# Supplementary figures and images for: Design of a novel filter paper based construct for rapid analysis of acetone
Source: PLoS One. 2018 Jul 6;13(7):e0199978. doi: 10.1371/journal.pone.0199978 (PMC6034825; doi:10.1371/journal.pone.0199978)

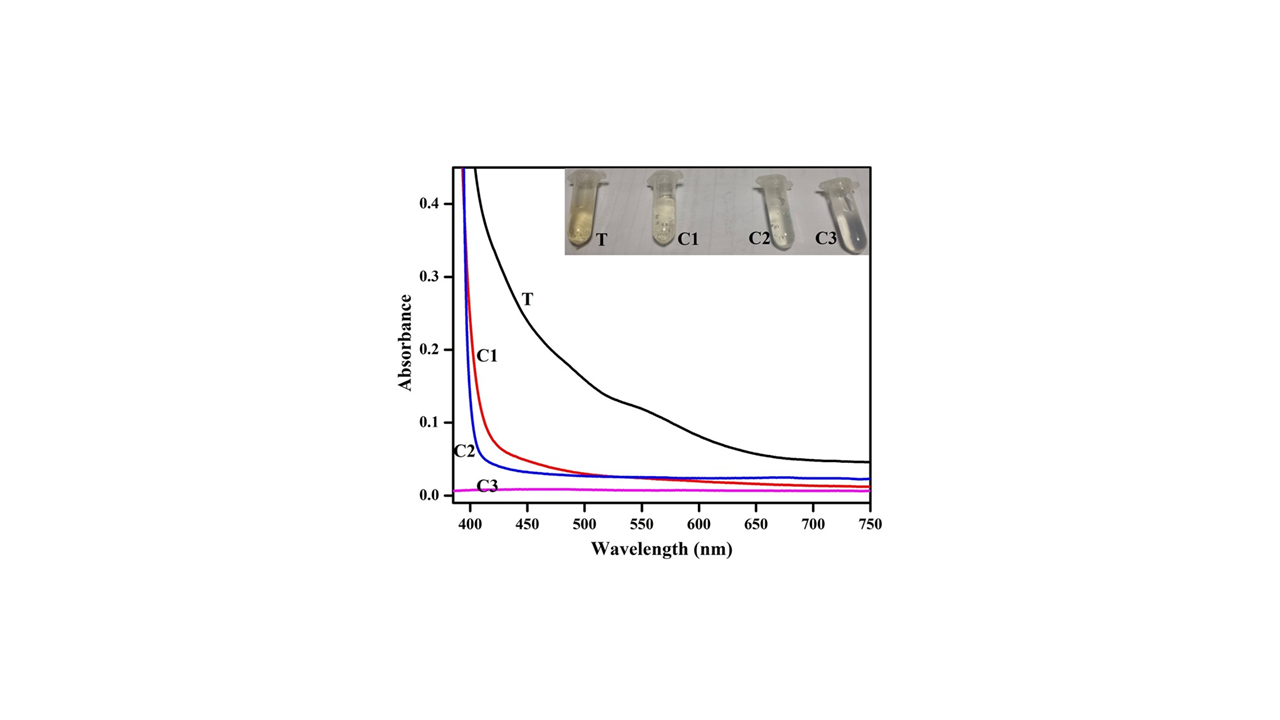

Supplement: S1 Fig — (TIF) [file pone.0199978.s001.tif]

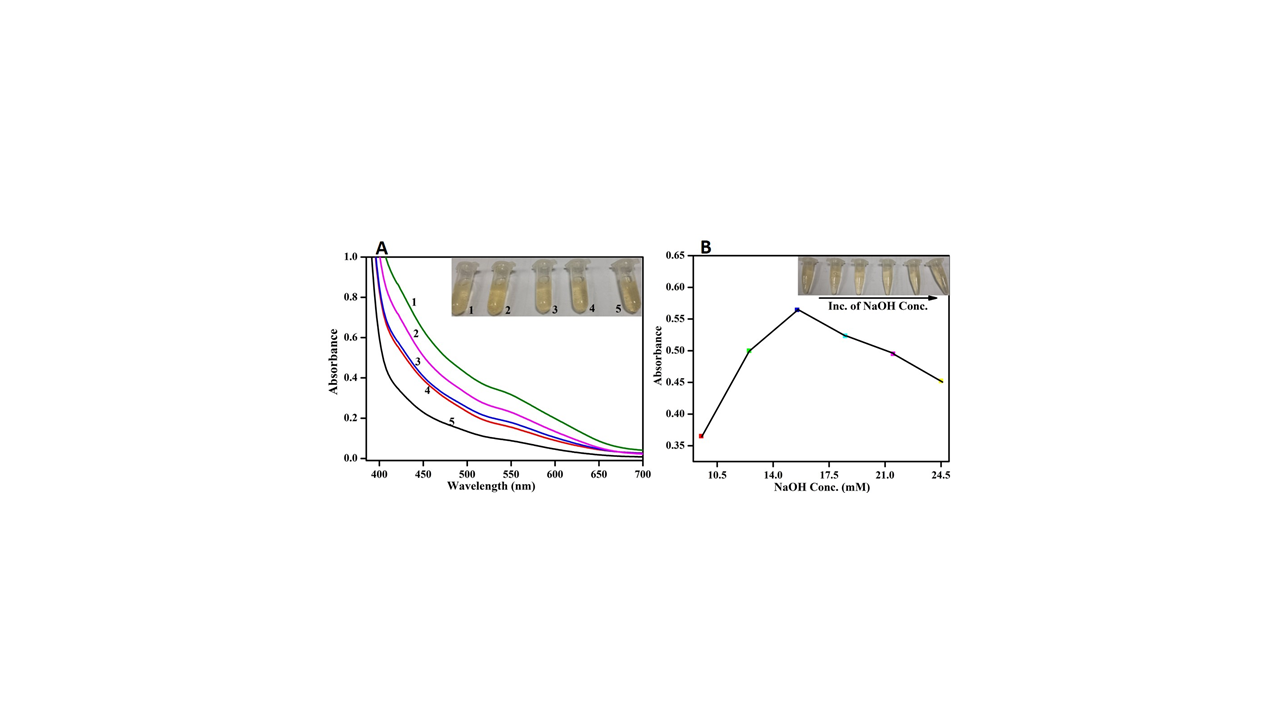

Supplement: S2 Fig — Optimization of volume of diazotized 4-aminobenzoic acid (μL), (1) 500, (2) 450, (3) 400, (4) 350, (5) 300 in the presence of NaOH (15.5 mM) and Acetone (0.25 mM) in 2 mL of de-ionized water reaction medium (A); Concentration of NaOH solution in the presence of 300 μL of diazotized 4-aminobenzoic acid and acetone (0.25 mM) in 2 mL of de-ionized water reaction medium (B). (TIF) [file pone.0199978.s002.tif]

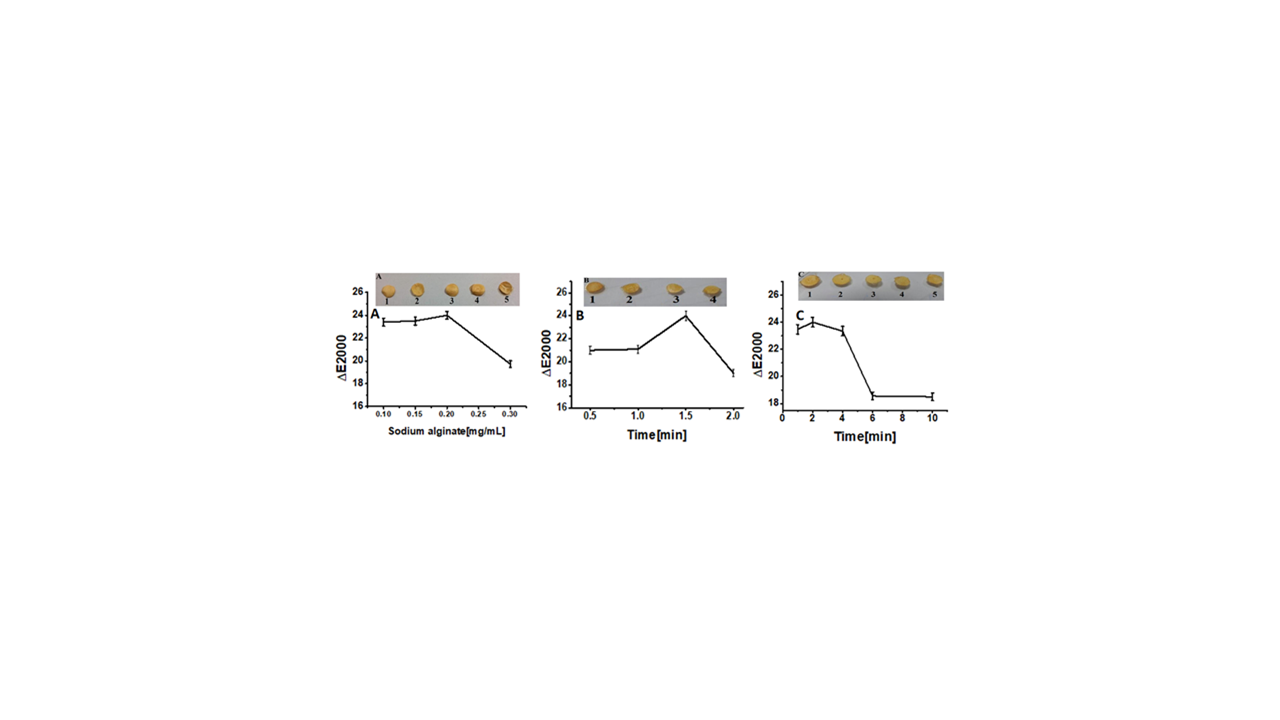

Supplement: S3 Fig — Optimization of concentration of amount of sodium alginate in gel (g/mL); (1) 0.1, (2) 0.15, (3) 0.2, (4) 0.25, (5) 0.3 (A); time of incubation of dipping filter paper strip to immobilize with modified sodium alginate gel (min); (1) 0.5, (2) 1, (3) 1.5, (4). 2 (B); time of incubation of acetone after addition on modified filter paper strip (min); (1) 1, (2) 2, (3) 4, (4) 6, (5) 10 (C). (TIF) [file pone.0199978.s003.tif]
