# Supplementary material for: Design of a novel filter paper based construct for rapid analysis of acetone
Source: PLoS One. 2018 Jul 6;13(7):e0199978. doi: 10.1371/journal.pone.0199978 (PMC6034825; doi:10.1371/journal.pone.0199978)
Supplement: S1 Table — (DOCX) [file pone.0199978.s004.docx]

Table. S1. Sensing data of the output color intensity and input concentration of acetone based on RGB color analyzing model.

| **S.No.** | **Acetone (ppm)** | **Color intensity** |
| --- | --- | --- |
| 1 | 10 | 215 |
| 2 | 20 | 206 |
| 3 | 40 | 197 |
| 4 | 80 | 192 |
| 5 | 160 | 187 |
| 6 | 320 | 164 |
| 7 | 640 | 152 |
