# Supplementary material for: Design of a novel filter paper based construct for rapid analysis of acetone
Source: PLoS One. 2018 Jul 6;13(7):e0199978. doi: 10.1371/journal.pone.0199978 (PMC6034825; doi:10.1371/journal.pone.0199978)
Supplement: S2 Table — (DOCX) [file pone.0199978.s005.docx]

Table. S2. Sensing data of the output color intensity and input concentration of acetone based on HSV color analyzing model.

| **S.No.** | **Acetone (ppm)** | **H value** |
| --- | --- | --- |
| 1 | 5 | 168 |
| 2 | 10 | 172 |
| 3 | 20 | 176 |
| 4 | 40 | 181 |
| 5 | 80 | 185 |
| 6 | 160 | 187 |
| 7 | 320 | 189 |
| 8 | 640 | 191 |
| 9 | 1000 | 194 |
|  |  |  |
