# Supplementary material for: Design of a novel filter paper based construct for rapid analysis of acetone
Source: PLoS One. 2018 Jul 6;13(7):e0199978. doi: 10.1371/journal.pone.0199978 (PMC6034825; doi:10.1371/journal.pone.0199978)
Supplement: S3 Table — (DOCX) [file pone.0199978.s006.docx]

Table. S3. Actual values of the input concentration and the output color intensity based on LAB color analyzing model.

| **S.No.** | **Acetone (ppm)** | **ΔE2000** |
| --- | --- | --- |
| 1 | 2.5 | 18.3 |
| 2 | 5 | 19.7 |
| 3 | 10 | 21 |
| 4 | 20 | 23 |
| 5 | 40 | 24.1 |
| 6 | 80 | 25 |
| 7 | 160 | 26.5 |
| 8 | 320 | 28.2 |
| 9 | 640 | 30 |
| 10 | 1000 | 30.5 |
| 11 | 1250 | 31 |
| 12 | 1500 | 32.17 |
